# Workforce Diversity and disparities in wait time and retention among opioid treatment programs

**DOI:** 10.1186/s13011-022-00500-3

**Published:** 2022-11-16

**Authors:** Erick G. Guerrero, Yinfei Kong, Jemima A. Frimpong, Tenie Khachikian, Suojin Wang, Thomas D’Aunno, Daniel L. Howard

**Affiliations:** 1 Research to End Healthcare Disparities Corp, I-Lead Institute, Los Angeles, CA USA; 2 Mihaylo College of Business and Economics, California State University, Fullerton, CA USA; 3 Business, Organizations and Society, New York University, Abu Dhabi, United Arab Emirates; 4 University of Chicago, Chicago, IL USA; 5 Department of Psychological and Brain Sciences, Texas A&M University, College Station, TX USA; 6 Wagner Graduate School of Public Service, New York University, New York City, New York USA

**Keywords:** Workforce diversity, Retention, Access, Healthcare disparities, Race, Opioid use disorder

## Abstract

**Background:**

Workforce diversity is a key strategy to improve treatment engagement among members of racial and ethnic minority groups. In this study, we seek to determine whether workforce diversity plays a role in reducing racial and ethnic differences in wait time to treatment entry and retention in different types of opioid use disorder treatment programs.

**Methods:**

We conducted comparative and predictive analysis in a subsample of outpatient opioid treatment programs (OTPs), who completed access and retention survey questions in four waves of the National Drug Abuse Treatment System Survey (162 OTPs in 2000, 173 OTPs in 2005, 282 OTPs in 2014, and 300 OTPs in 2017). We sought to assess the associations between workforce diversity on wait time and retention, accounting for the role of Medicaid expansion and the moderating role of program ownership type (i.e., public, non-profit, for-profit) among OTPs located across the United States.

**Results:**

We found significant differences in wait time to treatment entry and retention in treatment across waves. Average number of waiting days decreased in 2014 and 2017; post Medicaid expansion per the Affordable Care Act, while retention rates varied across years. Key findings show that programs with high diversity, measured by higher percent of African American staff and a higher percent of African American clients, were associated with longer wait times to enter treatment, compared to low diversity programs. Programs with higher percent of Latino staff and a higher percent of Latino clients were associated with lower retention in treatment compared with low diversity programs. However, program ownership type (public, non-profit and for-profit) played a moderating role. Public programs with higher percent of African American staff were associated with lower wait time, while non-profit programs with higher percent of Latino staff were related to higher retention.

**Conclusions:**

Findings show decreases in wait time over the years with significant variation in retention during the same period. Concordance in high workforce and client diversity was associated with higher wait time and lower retention. But these relations inverted (low wait time and high retention) in public and non-profit programs with high staff diversity. Findings have implications for building resources and service capacity among OTPs that serve a higher proportion of minority clients.

**Supplementary Information:**

The online version contains supplementary material available at 10.1186/s13011-022-00500-3.

## Background

The opioid epidemic disproportionately affects the nation’s 100 million low-income Americans and those who lack health insurance — groups that are disproportionately comprised of minority communities. Delivering culturally responsive care is critical to engage minorities in opioid use disorder (OUD) treatment and abate the effect of the epidemic. To identify key culturally competent practices that impact access and engagement (i.e., wait time to treatment entry and retention in treatment), it is necessary to examine the role of workforce diversity, one of the most concrete organizational culturally competent practices (i.e., matching staff-client culture, language, worldview based on racial/ethnic background) [1–3].

It is well established that individuals self-identified as members of racial and ethnic minority groups are more likely than White individuals to experience difficulty entering and staying in outpatient substance use disorder (SUD) treatment [4–6]. Reflecting their importance to treatment, wait time and retention have been included as performance measures developed by the Washington Circle [7], the Network for the Improvement of Addiction Treatment [8], and county and state administrative data systems [9]. Treatment access (measured through wait time to initiate treatment) and retention in treatment are critical process measures to abate disparities in substance use disorder treatment [10]. Yet, there is limited understanding of the role of workforce diversity in access and retention among OTPs. The following study addresses a significant gap in the cultural competency literature by examining the impact of workforce diversity, an essential culturally responsive practice, on disparities in treatment. We examine the impact of workforce diversity on quality of care in OTPs, measured by wait time and retention for individuals self-identified as Black/African American (hereafter African American) or Hispanic/Latino (hereafter Latino).

### Disparities in wait time and retention

The extant literature has established that wait time to enter SUD treatment is one of the most common challenges among those seeking help [11]. Particularly, among clients who self-described as African American or Hispanics, wait time is generally higher compared with clients self-described as non-Hispanic White [12–14]*.* Equally robust in the literature, treatment retention (i.e., time spent in treatment) is one of the most important predictors of reduced post-treatment substance use [7]. As healthcare reform has supplied new incentives for increasing access to and engagement in OUD treatment via Medicaid reimbursements, evaluating treatment access and retention within the context of Medicaid expansion is critical to responding to “treatment dropout” [15–17].

Developing understanding of the role of the Medicaid expansion on wait time and retention is critical in many ways. The main motivation of Medicaid expansion was to enhance access to care [13], hence it is important to examine how expected increases in public funding is associated with client treatment access and retention [18, 19]. Medicaid expansion also increases regulation on quality of care, that includes provision of culturally responsive and evidence-based SUD treatment [20]. Overall, Medicaid expansion may play a significant role in the delivery of culturally responsive care, as well as in wait time and retention.

### Workforce diversity and wait time and retention

Cultural competence is a set of behaviors and policies that enable a system, organization, or individual to function effectively with diverse clients and communities [21]. It also encompasses the culturally and linguistically appropriate services (CLAS) denomination used by federal health agencies [22, 23]. Workforce diversity is one of the six core components of CLAS: Leadership, Quality Improvement and Data Use, Workforce, Patient Safety and Provision of Care, Language Services, and Community Engagement.

Among racial/ethnic minorities, federal, state, and professional organizations have promoted cultural competence as a means to improve SUD treatment retention. Medicaid payments and related regulation have increased the focus on delivering services that respond to the cultural and linguistic services needs of clients [23]. The National Institute of Medicine, National Institute of Nursing and the National Association of Social Workers have all promoted workforce diversity strategies and developed training standards for cultural competency [24–26]. Regulation at the federal, state and professional certification levels have combined cultural competence in health care services [27–29]. Of particular relevance to the proposed research, the Substance Abuse and Mental Health Services Administration has called on providers to rely on CLAS because the majority of SUD counselors are non-Hispanic Whites [30, 31], even as 36% of clients at publicly-funded SUD treatment centers are non-White [32].

Following the work of Brach and Fraser, we define workforce diversity as the demographic and cultural representation of health workers and managers that reflect inclusion of backgrounds that are representative of the client population [33]. A workforce that represents client diversity is thought to be one of the main mechanisms to improve cultural and linguistic responsiveness.

However, the relationship between diversifying the workforce and disparities in access and retention in the SUD treatment system is not clear. Because culturally competent practices include a wide array of program arrangements, practices, and services, it is critical to determine which components of CLAS are most needed to engage minorities in OUD treatment. Some work shows that discordance between the racial and the ethnic diversity of clients and treatment staff widens healthcare disparities [2, 34, 35], while other work suggests that congruence is associated with disparities [36]. Congruence between the cultural and the linguistic backgrounds of staff and clients is thought to elevate the competencies of health care providers and improve client treatment adherence via the understanding of racial/ethnic history and cultural norms, as well as the communication through the client’s native language [37, 38]. This is thought to create a conducive climate for implementing CLAS (e.g., family support groups in Spanish) [39] and addressing the disparities in treatment outcomes among minorities [40, 41].

The field of SUD treatment has seen increased client diversity, yet limited longitudinal research has explored the provider/client similarity in racial/ethnic background [2, 5, 30]. In this exploratory work, we expect the role of workforce diversity to be associated with lower wait time and higher retention in well-resourced programs, while high diversity in staff and clients may be associated with higher wait time and lower retention in lower-resourced programs.

## Methods

We relied on data from the National Drug Abuse Treatment System Survey (NDATSS). This dataset contains eight waves of surveys of outpatient substance use disorder (SUD) treatment programs conducted in 1988, 1990, 1995, 2000, 2005, 2011, 2014, and 2017 [42–44]. Each survey wave since 1988 included programs from prior waves except for those dropped due to closure. Replacement programs were added to ensure adequate sample size. Representative samples of newer programs were added to keep the NDATSS representative of the changing population of US SUD treatment programs, including opioid treatment programs (OTPs). In this paper, we analyzed four waves of NDATSS data, focusing on pre- and post- Affordable Care Act Medicaid expansion (2000, 2005, 2014, and 2017) among OTPs that responded to survey questions on treatment access and retention. The analytic sample included: 162 OTPs in 2000, 173 OTPs in 2005, 282 OTPs in 2014, and 300 OTPs in 2017.

### Dependent variables

We considered two dependent variables in this study: 1) wait time, defined as average number of waiting days to begin treatment, 2) retention, defined as percent of clients remaining in treatment for more than 3 months. These are two well-established measures of engagement in the SUD treatment field [1–5]. Measuring the number of days to initiate treatment is associated with national standards (HEDIS performance measures [45]), while 90 days in treatment is well known, as a minimal exposure to treatment to show an effect [46].

### Independent variables

We included two workforce diversity variables: percent of African American staff and percent of Latino staff. Whether the treatment program resided in a state that expanded Medicaid was added as a control. Additional client and program characteristics relevant to our outcomes were also included: percent of African American clients, percent of Latino clients, percent of unemployed clients, accreditation by The Joint Commission (TJC), whether the treatment program was owned by a hospital, type of program (private for-profit, private not-for-profit, public), client-to-staff ratio, and proportion of staff with graduate degrees and total number of clients served in the previous fiscal year.

#### Statistical analysis

We conducted a comparative analysis of all variables across years. Chi-square tests or Analysis of Variance (ANOVA) were used to detect statistical differences across years. Since all dependent variables are continuous, we conducted linear regression analysis with normalized weight within each year to study the association of workforce diversity and other variables with the two dependent variables. We used weights to be consistent with analysis of NDATSS in other studies, even when the sample for the wait time analysis was limited to 198 programs and the sample for retention was limited to 661 of the 917 programs in the full sample. We conducted further analysis to identify sources of non-response bias, including missingness. Using these analytic samples, we examined the potentially moderated effect of workforce diversity and program type and clients’ race/ethnicity on wait time and retention.

## Results

### Differences in wait time and retention overtime

The comparative analysis across four waves is presented in Table 1. We found that mean waiting days were significantly different by year. More specifically, the mean waiting days decreased in the last two waves (post Medicaid expansion; *p* < 0.05). The percent of clients remaining in treatment more than 3, and more than 9 months were significantly different across years as well. Compared to the year 2000, the percent of clients treated more than 3 months was higher in 2005 and decreased in 2014 and 2017 (*p* < 0.01). In contrast, in 2000 the percent of clients treated more than 9 months was much higher in subsequent years (*p* < 0.01).

**Table 1:**
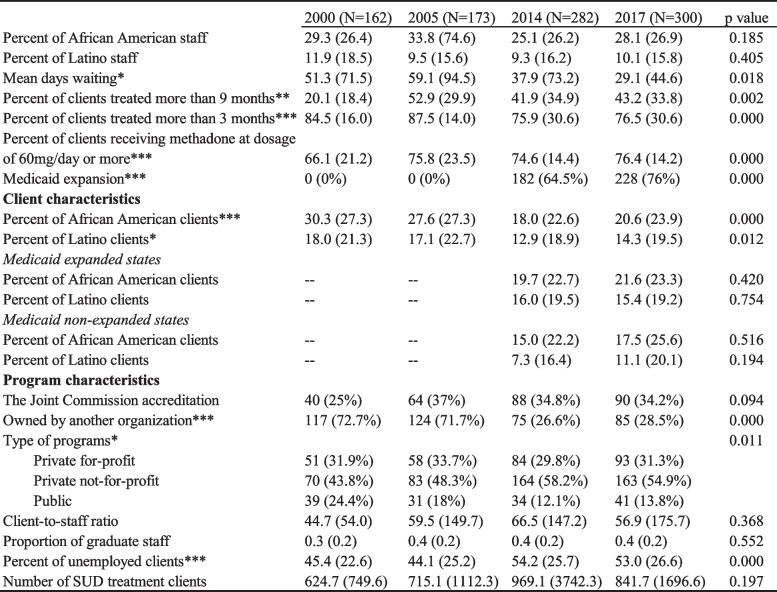
Comparative analysis of opioid treatment programs in NDATSS data

There were differences across years for program characteristics. For instance, programs owned by another organization were significantly different across years with a decrease in such programs in the years 2014 and 2017 (*p* < 0.01). Types of programs were also significantly different by year. There were more private not-for-profit programs in 2014 and in 2017, relative to public programs, which were more prevalent in 2017 (*p* < 0.05). Percent of unemployed clients was significantly different by year and higher in 2014 and in 2017 (*p* < 0.01).

### Workforce Diversity and wait time

Table 2 shows that programs with higher proportion of African American staff and higher proportion of African American clients were associated with higher mean waiting days compared with programs with lower proportion of African American clients and staff (beta = 0.015, *p* < 0.05). However, public programs with a higher percentage of African American staff were associated with lower mean waiting days compared to counterparts (i.e., private programs with lower percentage of African American staff; beta = − 1.328, *p* < 0.05). Similarly, public programs with higher percentage of Latino staff were associated with lower mean waiting days compared to counterparts (beta = − 5.073, *p* < 0.05).

**Table 2:**
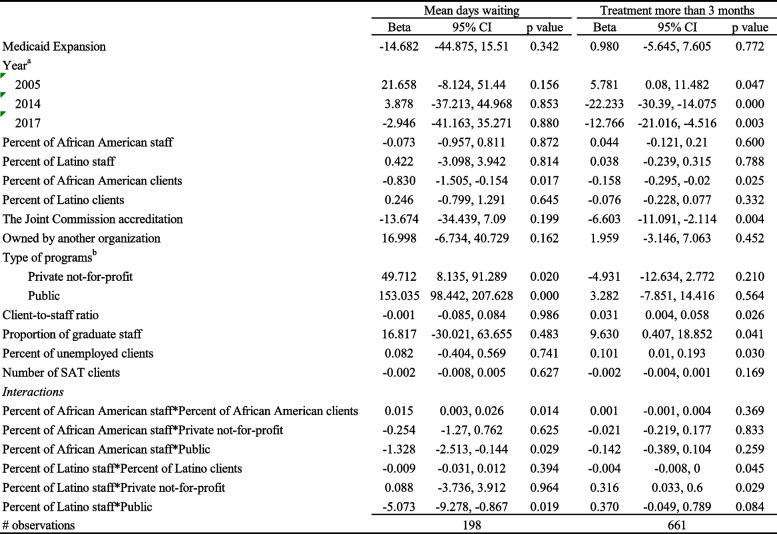
Linear regression models (with normalized weights & interactions)

### Workforce Diversity and retention

The programs with both a higher percentage of Latino staff and a higher percentage of Latino clients were associated with lower rates of treatment retention beyond 3 months (beta = − 0.004, *p* < 0.05). However, private not-for-profit programs with a higher percentage of Latino staff reported higher rates of treatment retention beyond 3 months (beta = 0.316, *p* < 0.05).

### Program structure factors and wait time and retention

Programs with higher percentage of African American clients were associated with lower average wait times (beta = − 0.830, *p* < 0.05) compared with programs with lower percent of African American clients. Regarding program ownership type, both private not-for-profit (beta = 49.710, *p* < 0.05) and public (beta = 153.035, *p* < 0.01) programs were associated with higher wait times compared to for profit programs. Client-to-staff ratio was positively associated with retention (percent of clients treated more than 3 months (beta = 0.031, *p* < 0.05. Retention was also positively associated with proportion of graduate staff (treatment staff with a masters and or doctoral degrees, beta = 9.630, *p* < 0.05) and percent of unemployed clients (beta = 0.101, *p* < 0.05).

## Discussion

We explored the association between workforce diversity and wait time to treatment entry and retention among a subsample of OTPs surveyed in 2014 and 2017 as part of the National Drug Abuse Treatment Services Survey (NDATSS). We found significant differences in wait time and retention across all four waves. Specifically, mean waiting days decreased in the last two waves (post Medicaid expansion), while retention rates varied across all years pre- and post-Medicaid Affordable Care Act expansion.

Key findings show that diversity in program staff and clients were associated with wait time and retention. Specifically, programs with higher percentage of African American staff and a higher percentage of African American clients were associated with more waiting days to enter treatment, compared to programs with lower percentages of African American staff and clients. Programs with both a higher percentage of Latino staff and a higher percentage of Latino clients stayed in treatment fewer days than programs with both a lower percent of Latino staff and clients. Consistent with other studies, these findings on the negative association between workforce diversity and measures of treatment engagement suggest that there may be other factors that explain these differences in access and retention [19, 47, 48]. Previous analyses of the NDATSS determined that treatment units with high client diversity tend to have lower retention rates as well as fewer engagement approaches to improve retention [49–51]. Albeit conjectural, programs with high percentage of African American and/or Latino staff and clients are often small, under resourced and challenged to implement innovative engagement practices [10, 52, 53].

These “high minority” programs also tend to be located in lower income and under-resourced minority communities [53–55]. Worse outcomes in highly diverse programs may therefore be explained by factors, such as low-capacity programs serving high-service need clients (clients with mental health, employment, and childcare service needs) [56]. This notion is supported by studies which have considered diversity and high program resources together and found that access and retention are generally high in the presence of both factors [19]. Our findings suggest that workforce diversity as a mechanism of cultural competence via staff-client racial/ethnic and language matching might be most effective when programs have the organizational policies, practices, training, and other resources to improve standards of care overall. This further draws upon the importance of including and defining core components of competence through organizational, resources, efforts and practices. Culturally and linguistically Appropriate Services (CLAS) have been associated with SUD treatment access and engagement [2, 10, 13]. These CLAS generally include offering translated material, bilingual/bicultural counselors, space and services for families, while responsive policies include accessible service hours, flexible service delivery approaches, accepting Medicaid [2, 10, 19].

Two critical findings highlight the importance of program type to maximize the role of workforce diversity on wait time and retention. We found lower wait time in public programs with high African American staff and high Latino staff, and higher retention in nonprofit programs with more Latino staff. Compared with for profit programs, public programs may have certain policies and practices that improve access to care, while non-profit may have policies and practices that keep people in needed care longer. As for-profit programs have grown the fastest across the nation, it is critical to further examine differences in workforce diversity and engagement approaches among these different program types.

We would like to recognize limitations of this study. First, bias related to program managers over-reporting positive features, such as lower wait times and greater retention may exist. We used auxiliary variables to reduce this bias and determine the most accurate program condition regarding OTP practices and services. Moreover, using existing data from real-world treatment settings may not be optimal for assessing our OTP process outcomes, but it is the most comprehensive and reliable source [57]. To determine its reliability, we compared NDATSS data on key outcomes obtained from client records in a national random sample of methadone treatment units in the National Evaluation of Substance Abuse Treatment Study [43]. Third, there have been substantive changes in the OTP environment since the last waves of our data (2017). The number of for-profit OTPs has expanded substantially and now represents about two-thirds of OTPs. Because our workforce diversity findings stress the importance of program type (public, non-profit and for profit) and regulation regarding access to take-home medication treatment is rapidly changing, our findings serve as baseline evidence in need of further exploration. Another limitation is that our analysis was limited to a sample of programs that responded to our access survey items. While our missing data analysis (see Appendix A) did not reveal differences in key characteristics between our analytic and full sample, our findings only represent a subsample of NDATSS opioid treatment programs. Findings based on this subsample are still important and provide a baseline understanding of the role of workforce diversity on treatment access and engagement in opioid treatment. NDATSS findings on the effect of program features on OTP process outcomes have been consistent with results from regional studies using program and client data [10, 58]. Lastly, our findings might be affected by nonrandom attrition of programs from the sample over time and unaccounted unobserved program characteristics affecting our outcomes. To mitigate bias related to these two conditions, we accounted for repeated observations from the same unit over time using random effects models. Despite these limitations, findings from our study are likely to inform policy and practice interventions tailored to the most common opioid treatment settings nationwide.

## Conclusions

Findings expand our understanding of the complex role of workforce diversity in enhancing access and retention in opioid treatment. It is critical to further examine workforce diversity within the potential resource and capacity needs of vulnerable OTPs serving minority communities. As federal and state authorities promise a significant influx of financial resources drawn from pharmaceutical settlements and new taxation revenues to enhance access to opioid treatment, it is critical to support OTPs in minority communities to improve treatment outcomes. The overall resources directed to opioid treatment at the federal level amount to $125 billion over ten years, promised by the Biden administration [59]. As the treatment field faces significant challenges in workforce training, diversity and the use of evidence-based practices, this financial resource can be more effectively directed towards research, training and support of the workforce. The delivery of culturally responsive and medication-assisted care to vulnerable populations suffering from OUD must be meaningfully integrated into such efforts. Leading federal agencies (e.g., National Institute of Health, Substance Abuse and Mental Health Services Administration, Center for Disease Control), with an emphasis on process and outcome improvements, have called attention to the aforementioned areas, in order to reduce the rate of overdose deaths, one of the toughest consequences of limited access to care.

## Supplementary Information

**Additional file 1:** **Appendix A.** Comparative analysis of missing records for wait time and retention

## Data Availability

The datasets used and/or analyzed during the current study are available from the corresponding author on reasonable request.

## Abbreviations

OTP: Opioid treatment program
NDATSS: National Drug Abuse Treatment System Survey
OUD: Opioid use disorder
SUD: Substance use disorder
CLAS: Culturally and linguistically appropriate services
TJC: The Joint Commission
N-SSATS: National Survey of Substance Abuse Treatment Services
