# Supplementary material for: Workforce Diversity and disparities in wait time and retention among opioid treatment programs
Source: Subst Abuse Treat Prev Policy. 2022 Nov 16;17:74. doi: 10.1186/s13011-022-00500-3 (PMC9670430; doi:10.1186/s13011-022-00500-3)
Supplement: Supplementary file 1 [file 13011_2022_500_MOESM1_ESM.docx]

Table 1A. Comparative analysis of missing records for mean days waiting

	Non-Missing (N = 198)	Missing (N = 719)	p value
Year			0.448
2000	32 (16.2%)	130 (18.1%)	
2005	44 (22.2%)	129 (17.9%)	
2014	55 (27.8%)	227 (31.6%)	
2017	67 (33.8%)	233 (32.4%)	
Percent of African American staff	33.1 (68.8)	27.0 (26.7)	0.217
Percent of Latino staff	9.1 (16.6)	10.3 (16.2)	0.346
Percent of African American clients	27.3 (27.9)	21.6 (24.2)	0.010
Percent of Latino clients	12.18 (18.21)	15.96 (20.92)	0.014
The Joint Commission accreditation	68 (34.3%)	214 (32.9%)	0.765
Owned by another organization	87 (43.9%)	314 (43.9%)	1.000
Type of programs[b]			0.000
Private for-profit	36 (18.2%)	250 (35.1%)	
Private not-for-profit	123 (62.1%)	357 (50.1%)	
Public	39 (19.7%)	106 (14.9%)	
Client-to-staff ratio	74.1 (239.3)	53.3 (103.2)	0.235
Proportion of graduate staff	0.3 (0.2)	0.4 (0.2)	0.575
Percent of unemployed clients	56.7 (23.8)	48.3 (26.0)	0.000

Table 1B. Comparative analysis of missing records for retention

	Non-Missing (N = 661)	Missing (N = 256)	p value
Year			0.000
2000	116 (17.5%)	46 (18%)	
2005	151 (22.8%)	22 (8.6%)	
2014	190 (28.7%)	92 (35.9%)	
2017	204 (30.9%)	96 (37.5%)	
Percent of African American staff	29.1 (44.0)	25.9 (25.4)	0.188
Percent of Latino staff	10.0 (16.5)	10.2 (15.4)	0.835
Percent of African American clients	23.6 (25.8)	20.6 (23.2)	0.133
Percent of Latino clients	15.53 (20.77)	13.39 (18.81)	0.186
TJC accreditation	204 (30.9%)	78 (41.5%)	0.008
Owned by another organization	300 (45.4%)	101 (39.9%)	0.157
Type of programs[b]			0.009
Private for-profit	226 (34.2%)	60 (24%)	
Private not-for-profit	330 (49.9%)	150 (60%)	
Public	105 (15.9%)	40 (16%)	
Client-to-staff ratio	60.6 (160.6)	49.5 (81.8)	0.197
Proportion of graduate staff	0.3 (0.2)	0.4 (0.2)	0.284
Percent of unemployed clients	49.8 (25.7)	51.8 (25.7)	0.353
